# High-Pressure Injection Injuries of the Hand: Our Experience and Literature Review

**DOI:** 10.1055/s-0045-1807242

**Published:** 2025-10-29

**Authors:** Anand Prasath Jayachandiran, Manoj Ananthappan, Vasireddy Divya, Surya Rao Rao Venkata Mahipathy, Alagar Raja Durairaj, Suresh Rajendran

**Affiliations:** 1 Department of Plastic and Reconstructive Surgery, Saveetha Medical College and Hospital, Thandalam, Tamil Nadu, India; 2 Department of General Surgery, Saveetha Medical College and Hospital, Thandalam, Tamil Nadu, India

**Keywords:** hand, high pressure, injection injury

## Abstract

**Background:**

High-pressure injection injuries are serious and often underestimated traumas that can lead to severe soft tissue damage, ischemia, infection, and even amputation. These injuries commonly occur in industrial settings, particularly affecting young male workers handling chemicals.

**Materials and Methods:**

Seven male patients with high-pressure hand injection injuries from November 2019 to December 2024 were evaluated retrospectively. Immediate debridement and broad-spectrum antibiotics were administered. Follow-up assessed the recovery and functional outcomes.

**Results:**

Out of seven patients, one was managed conservatively and the others required multiple debridements, with two presenting with infections and one with compartment syndrome. X-rays confirmed the presence of foreign bodies in all cases. Soft tissue defects were addressed using reconstructive techniques. One patient developed finger stiffness, and two were lost to follow-up. In most cases, aggressive early intervention and debridement reduced infection and preserved hand function, although some patients experienced prolonged recovery due to tissue loss and the need for flap coverage.

**Conclusion:**

High-pressure injection injuries require rapid recognition and management, as delays increase the risk of infection, necrosis, and functional impairment. Early, thorough debridement is essential, especially with materials like paint thinner significantly elevating the risk of amputation. This study emphasizes educating emergency physicians on early recognition and intervention for optimal outcomes.

## Introduction

High-pressure injection injuries may appear as tiny puncture wounds but can cause severe consequences that spread subcutaneously from the fingertip to the mediastinum. 1 These injuries are frequently caused by high-pressure cleaning equipment misfires or leaks, which pump fluids such as oil, diesel, paint, and solvents into the hand at up to 10,000 psi. 2 The material's high velocity allows it to penetrate deeply, spreading via fascial planes and possibly reaching the forearm and proximal hand. 3 These compounds can cause severe harm through ischemia, mechanical impact, infection, and elevated pressure within the hand's confined spaces, resulting in compartment syndrome. 2 High-pressure injection injuries account for 1 in every 600 cases of hand trauma in emergency rooms, with young male industrial workers handling chemicals like paint and lubricants being most at risk. 4 The nondominant index finger is commonly affected, though the thumb, palm, and other extremities are also vulnerable. 5 Radiopaque materials can be found on radiographs, but radiolucent substances can only be found by other signs, such as subcutaneous emphysema. Soft tissue injury can be seen using CT and MRI scans. However, the mainstay of treatment is early and extensive surgical debridement. 6 Amputation or loss of function can only be avoided by surgical intervention, which includes compartment decompression, lavage, and removal of injected materials. 7 Although high-pressure injection injuries have been well documented, our study provides new insights into the role of early aggressive debridement and its effect on infection control. We also analyze cases where neurovascular preservation was attempted despite paint embedding in the fascial planes

## Materials and Methods

A departmental retrospective evaluation was performed between November 2019 and December 2024. All patients who underwent high-pressure injection injury management were analyzed. Age, gender, finger affected, number of debridements, wound management, and follow-up were all recorded. The nature of the injected substance, associated comorbidities, time gap from injury to presentation, microbiology evaluation reports in needed patients, and radiology reports were all analyzed. All the details were categorized and tabulated. A literature review was also conducted, and our experience was compared.

## Results

Our study included seven male patients aged 19 to 32 years. Out of them, five had left-hand injuries. Six patients got paint as the injected material, and one patient presented with air being injected under high pressure. Three of the patients presented late, about a week after the original injury, and each patient required numerous debridements. Following debridement, two of these late presentations experienced soft tissue loss ( Table 1 ). Two individuals had infection-related symptoms, and *Pseudomonas* was found in their cultures. A patient came with finger compartment syndrome. All patients with paint injections were debrided on the day they arrived at the department and put on broad-spectrum antibiotics to cover gram-positive and gram-negative bacteria under the institutional infection control protocol. Three patients with soft tissue defects received coverage using cross-finger and abdominal flaps, two underwent delayed primary closure, and one underwent shortening with cross-finger flap cover. Postoperative physical therapy was initiated for all patients. One patient was lost to follow-up, and another developed finger stiffness; all returned to the same job. The patient's air injection was managed conservatively with limb elevation and antibiotics.

**Table 1:** Assessed parameters

Sl. no.	Age/sex	Involved finger and dominance	No. of days after injury	No. of debridements	Wound management	Remarks
1	19/M	Right middle, D	0	3	Cross-finger flap	Culture positive
2	29/M	Left index, ND	10	2	Secondary suturing	–
3	19/M	Left ring, ND	0	2	Shortening with cross-finger flap	Compartment syndrome at presentation
4	27/M	Left index, ND	7	2	Cross-finger flap	Lost to follow-up
5	32/M	Left palm over the third MCP joint, ND	0	2	Secondary suturing	–
6	24/M	Left ring and middle, ND	6	3	Abdominal flap	Stiffness of left ring finger Culture positive
7	23/M	Right first web, D	0	0	Conservative	–

Abbreviations: D, dominant; MCP, metacarpophalangeal; ND, nondominant.

## Representative Cases

**Fig. 1:**
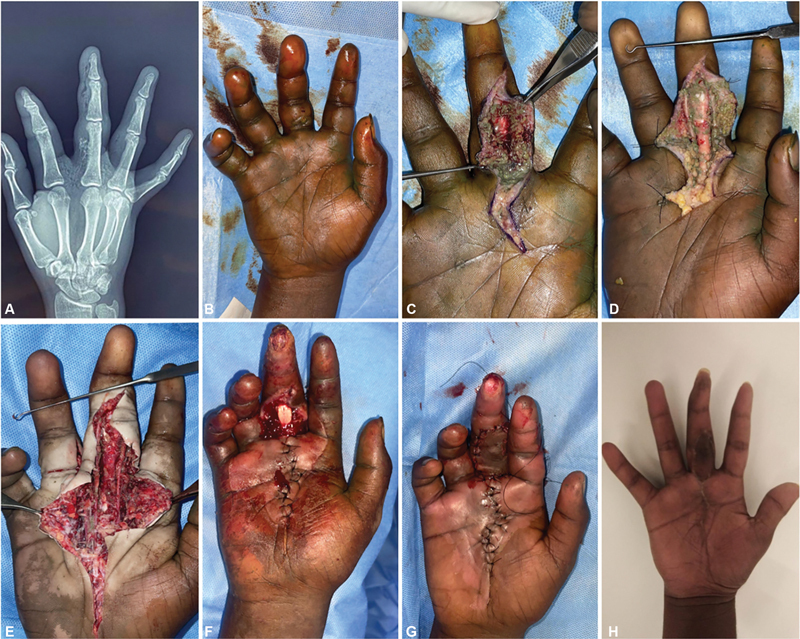
( **A,B** ) Preoperative X-ray and injured hand. ( **C,D** ) First debridement showing paint lodged in subcutaneous tissue and around neurovascular bundle. ( **E** ) Second extensive debridement. ( **F,G** ) Third debridement and same-sitting cross-finger flap coverage. ( **H** ) Late follow-up.

**Case 1:** A 19-year-old man presented with an injury to his right hand sustained while handling a paint gun. He experienced swelling, pain, and a puncture wound on the volar side of the middle finger near the palmar crease. X-rays showed a foreign object extending to the third metacarpophalangeal (MCP) joint. The patient underwent three debridements. The paint was lodged in the subcutaneous plane, adhering to the neurovascular bundle. The patient had a positive culture and was treated with appropriate antibiotics. A cross-finger flap was applied on postoperative day 14. Subsequently, on follow-up, the patient developed a finger deformity ( Fig. 1 ).

**Fig. 2:**
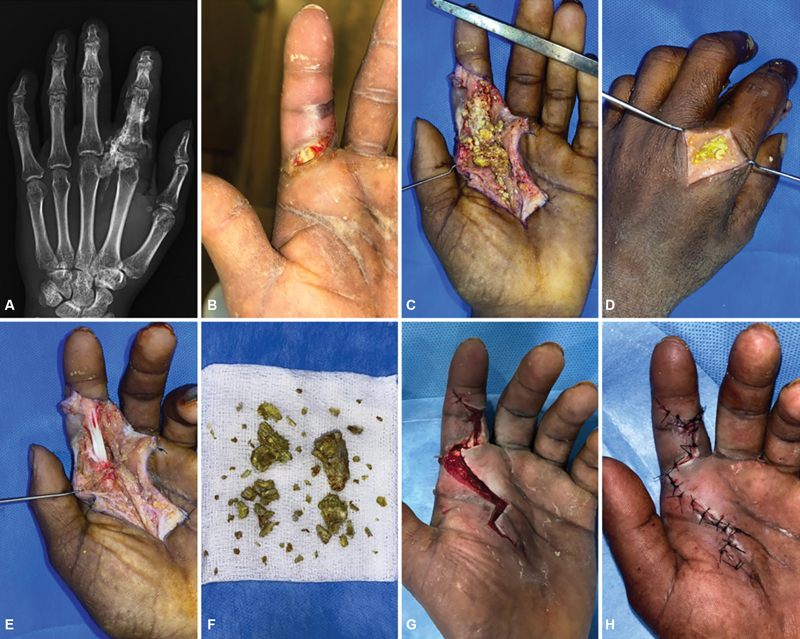
( **A,B** ) Preoperative X-ray and injured hand. ( **C,D** ) First debridement showing solidified paint around flexor tendon sheath and dorsum of hand. ( **E,F** ) After complete removal of foreign body. ( **G,H** ) Second debridement and secondary closure on the same sitting.

**Case 2:** A 29-year-old man arrived with ulceration and tenderness in the left index finger. The patient had sustained an injury 10 days back while using a paint gun. He underwent debridement elsewhere on the day of the incident. X-rays showed foreign objects extending from the second web space to the second MCP joint and proximal phalanx. The paint was lodged in the subcutaneous plane, adhering to the neurovascular bundle, spread along the flexor tendon sheath and extended to the dorsal aspect. He underwent two debridements, and the wound was closed secondarily immediately after the second debridement. Postoperative physical therapy was started to enhance recovery. Follow-up showed good functional recovery ( Fig. 2 ).

**Fig. 3:**
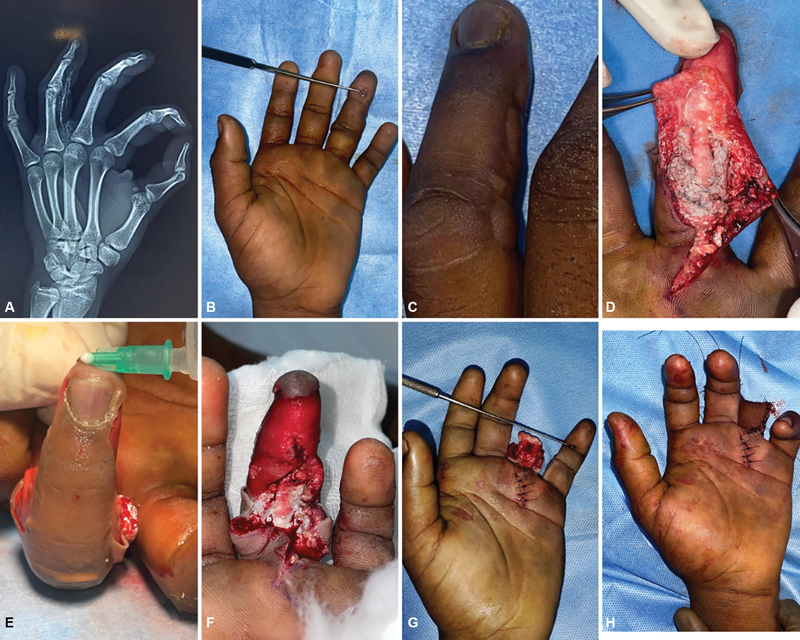
( **A,B** ) Preoperative X ray and injured hand. ( **C** ) First debridement showing injured ring finger with blisters in radial side. ( **D** ) Paint lodged around the neurovascular bundle and erythema in the distal finger. ( **E** ) Congested and discolored finger immediate post-op. ( **F** ) Postoperative day 1 showing discolored tip and erythema. ( **G,H** ) Postshortening of the finger and cross-finger flap coverage.

**Case 3:** A 19-year-old man presented with an injury to his left middle finger with blisters on both the ulnar and radial aspects, edema, and a puncture wound on the volar surface over the proximal interphalangeal joint. He sustained the injury while using a paint gun. The patient was diagnosed with impending finger compartment syndrome. X-rays showed a foreign body in the volar aspect of the left ring finger. The paint was found to be lodged around both radial and ulnar neurovascular bundles with erythema in the distal finger. The patient had debridement, after which the finger was found unsalvageable 2 days later. The patient requested that the stump be preserved as much as possible. A second debridement was done to shorten and cover the volar defect with a cross-finger flap from the left middle finger ( Fig. 3 ).

**Fig. 4:**
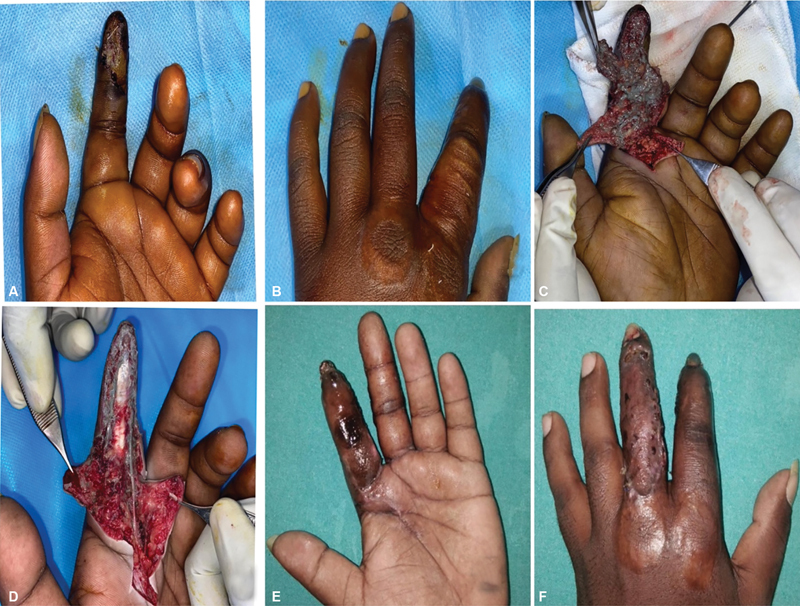
( **A,B** ) Preoperative. ( **C** ) First debridement showing diffuse spread. ( **D** ) Second debridement showing spread along the neurovascular bundle. ( **E,F** ) Following cross-finger flap coverage.

**Case 4:** A 27-year-old man presented with an injury to his left index finger. The patient reported that the injury occurred 1 week prior when a paint gun accidentally fell on his hand. He was initially treated elsewhere. On examination, the left index finger exhibited discolored volar skin, swelling, and tenderness around the MCP joint. The injected paint was found to be diffusely spread along the subcutaneous plane, extending to the palm along the neurovascular bundle. The patient underwent two debridements, and the defect was covered with a cross-finger flap. Postoperatively, the patient was started on physical therapy to aid recovery ( Fig. 4 ).

**Fig. 5:**
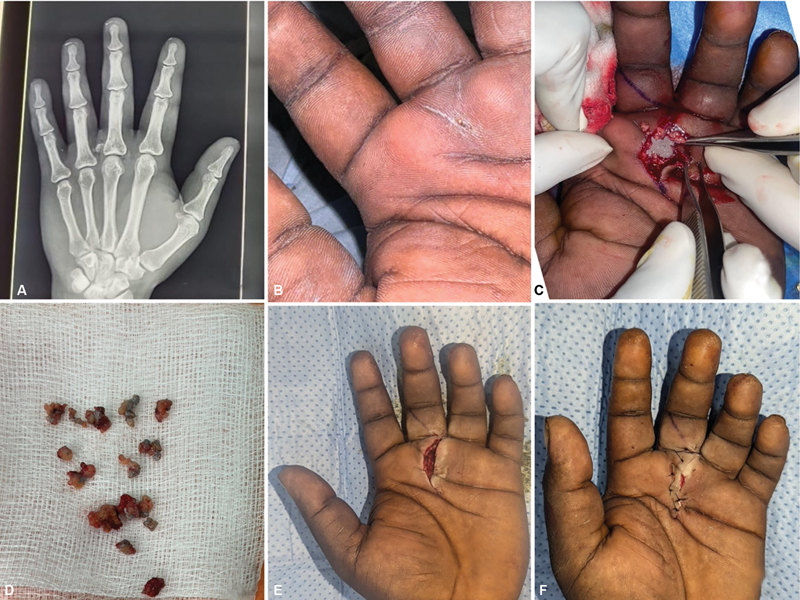
( **A,B** ) Preoperative X-ray and point of entry. ( **C** ) First debridement showing paint. ( **D** ) Evacuated paint material. ( **E,F** ) Pre- and post-op of secondary suturing.

**Case 5:** A 32-year-old man presented with injury to the left palm near the third web space at the workplace while using a paint gun. He arrived at the emergency room a few hours after the injury. There was tenderness at the site on examination, and X-ray imaging revealed a radiopaque foreign body. The paint was lodged only in the subcutaneous plane. The patient underwent debridement and was started on antibiotics. Secondary suturing was performed after a week of wound dressing ( Fig. 5 ).

**Fig. 6:**
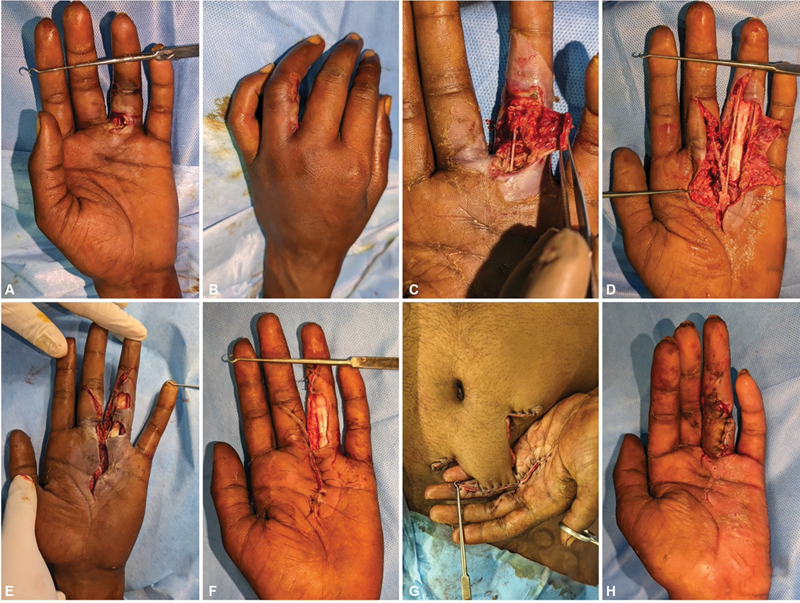
( **A,B** ) Preoperative injured hand. ( **C,D** ) First debridement showing tracking of material along the neurovascular bundle. ( **E,F** ) Second debridement and secondary suturing with raw area. ( **G** ) After abdominal flap coverage. ( **H** ) After flap division.

**Case 6:** A 24-year-old man presented with a workplace injury to his left hand sustained 6 days prior while operating a paint gun. On examination, there was a raw area with pus discharge extending from the proximal to the digital palmar crease to the proximal phalanx, with decreased sensation over the left middle and ring fingers. Debridement and foreign body removal revealed paint adherent to the ulnar neurovascular bundle of the middle finger and the radial neurovascular bundle of the ring finger, extending proximally to the palmar crease and proximal phalanx. The patient underwent further debridements, followed by abdominal flap coverage and primary skin suturing to manage the defect ( Fig. 6 ).

**Fig. 7:**
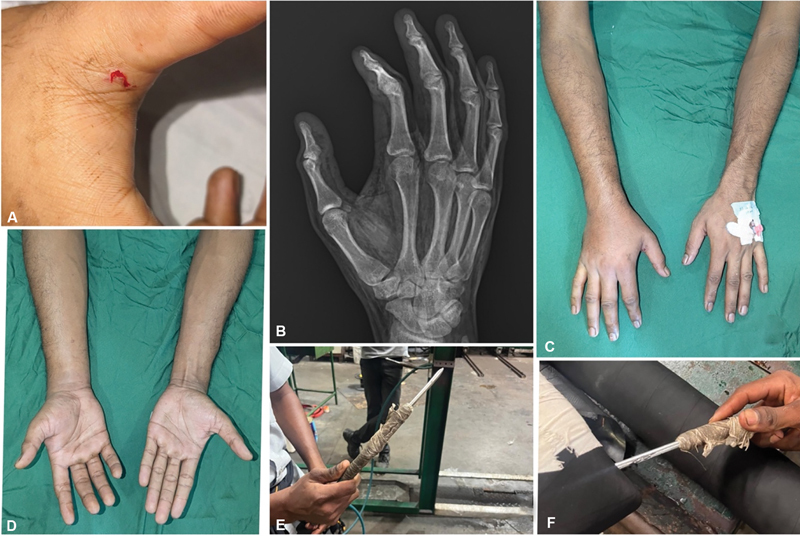
( **A** ) Preoperative point of entry. ( **B** ) X-ray showing subcutaneous emphysema. ( **C,D** ) Limb size discrepancy—right side edematous compared to the left. ( **E,F** ) Source of injury and its application.

**Case 7:** A 23-year-old man presented with a workplace injury to his right hand caused by an air compression needle. On examination, a puncture wound was observed at the first web space, with crepitus extending from the right hand to the axilla. X-ray imaging revealed subcutaneous emphysema. The patient was managed conservatively with limb elevation and compression dressing. His condition improved within 2 days without complications. On review, the patient had no functional deficit ( Fig. 7 ).

## Discussion

High-pressure injection injuries can cause both mechanical and chemical trauma, often affecting deep tissues such as the neurovascular bundle, tendon sheath, or along any fascial planes/plane of least resistance, which may vary in each patient depending on the material, pressure, and duration of contact. According to Kaufman, high-pressure materials diffuse throughout the neurovascular bundle until they come into contact with resistant tissues, at which time they change course. 8 In our series, the substance was traveling along the neurovascular plane in five patients and one along the tendon sheath, which also extended to the dorsal surface.

Caustic chemicals directly irritate tissues and result in potential amputations, necrosis, and ischemia. 9 10 To decrease/prevent the injury leading to any complications (infection/compartment syndrome/tissue loss/amputation), we prefer early debridement and prophylactic antibiotics. Debridement should be extensive, and extending incisions proximally and distally is better till normal tissue is seen. A low threshold should be kept for extending the incisions, as removing the injected materials helps with wound closure and infection prevention.

It is advised to do debridements under regional blocks as we may not know the extent of the spread of the injected materials.

Pathogens such as *Clostridium* , β-hemolytic streptococci, and *Staphylococcus aureus* are known to produce infections, which pose a significant threat since they can worsen tissue damage and raise the risk of systemic disease. 11 In our series, one patient had an identifiable pathogen, *Pseudomonas* , causing infection. The patient was put on appropriate antibiotics and underwent flap cover.

As in the literature, most of our patients did not have an organism identified from the wound. This could be due to the early use of prophylactic antibiotics.

In the study by Nichols et al, middle-aged male workers are the group most commonly affected by high-pressure injection accidents. Similarly, our study included five male patients, aged 19 to 32 years, with injuries primarily to their left nondominant hand. Based on our experience, the most commonly injured fingers were the index and middle fingers. 12

Every case in our analysis resulted from exposure to pressure guns with mostly paint injections and one with air injection at work. Chemicals, including paint thinners, industrial solvents, and oil-based paints, damaged tissue more severely than chemicals based on water. Oil materials stay in tissues, causing chemical irritation and chronic inflammation. The amputation rate for oil-based paint-related injuries is approximately 50%, as reported by Mirzayan et al 13 and Yıldıran et al. 14 They have suggested early vascular blockage resulted in ischemia and necrosis leading to amputation. In our series, we had one patient who presented with a finger compartment with a compromised vascular supply shoe and ended up in amputation. We suggest early debridement and using Brunner's incision to release the finger compartment, as it will also help remove the chemical. A mid-lateral incision, though it may decompress the finger wounds, is adequate for removing the injected materials. In contrast, no amputations have been reported in studies with air and water injections, making them less dangerous. 10

The force with which the substance is delivered significantly influences the degree of the damage. Complete debridement becomes more complex as pressure increases, causing deeper material penetration and tissue dispersion. This increases the risk that residual material may cause additional harm after the initial debridement. Extensive debridement is advised, and it should be gauged against the vascularity of the digit, as it may revascularize the digit. If there is doubt of vascularity, serial frequent debridements can be planned.

Knowledge about this type of injury (in the emergency department) is essential to avoid delays in the treatment and prompt referrals. The entry point will be small and must be diagnosed with high suspicion. History is the primary diagnostic of high-pressure injection injury; no specific investigation is required for diagnosis. Emergency therapy includes tetanus prophylaxis, wound irrigation, and broad-spectrum antibiotics to avoid infection. Preventative antibiotics, such as third-generation cephalosporins, are crucial to lowering the risk of infection, especially while handling polluted industrial materials. Using Ringer's lactate as an irrigation solution has been suggested to help remove foreign objects and lessen inflammation. 9 10 Furthermore, radiopaque materials show up on X-rays, making it easier to assess the level of debridement and guarantee that no foreign bodies are overlooked. An extensive anamnesis and a thorough neurovascular evaluation are necessary to determine the degree of the injury and plan the surgery.

In the case of radiolucent materials, CT and MRI have been used to study the extent of tissue injury. We have no experience with such modalities. We had one patient who was injected at high pressure, and the extent of the injury was evident based on subcutaneous emphysema.

In our experience, most of the injected material travels along the neurovascular bundle if the site of injections is at the finger level. Early extensive debridement is the key to decreasing morbidity and improving functional outcomes. Serial and frequent examination of the wound is vital. There should be a low threshold for further debridement. Exposed tendons can be covered with collagen sheets to decrease desiccation until the wound is covered.

According to Stark et al, 7 15 delaying treatment for more than 10 hours significantly increases the risk of amputation. The best results are seen when debridement is performed during the first 6 hours. 9 In our experience, we could not find any correlation between the outcome and time of presentation after injury. Both early and late presenters had tissue loss and underwent flap cover.

Although it is advocated to ensure no leftover chemicals, 10 we advise not to explore or dissect between the digital nerve and artery. Sometimes, removing the substance encircling the neurovascular bundle may be impossible. In the scenarios in which the substance encircles the neurovascular bundle, the viability of the digit should be weighed more, and debulking of the substance rather than complete removal can be done. Even if it is the last option, amputation could occasionally be required to save the patient from a severe functional impairment or persistent infection.

## Conclusion

High-pressure injection injuries are surgical emergencies that need to be recognized immediately and evaluated by a hand surgeon as soon as possible. Emergency physicians need to be educated more than anybody since prompt action improves patient outcomes. Early, extensive debridements with frequent vigilant observation of the wound with a low threshold for serial debridements are the key in our experience for better functional outcomes. Early wound coverage should be done once the wound shows signs of healing and no signs of infection or proximal extension. Additionally, our experience suggests that neurovascular dissection should be limited when injected material surrounds vital structures, as complete removal may not always be necessary for favorable outcomes.
